# Antioxidative and Anti-Inflammatory Mechanisms of Quercetin in the Prevention of Secondary Diabetes Complications

**DOI:** 10.3390/ijms27209315

**Published:** 2026-10-20

**Authors:** Alyssa Uribe, Brandon Kassel, Kaleb J. Maher, Wyatt C. Parry, Ahmad N. Amanullah, Kota V. Ramana

**Affiliations:** Department of Biomedical Sciences, Noorda College of Osteopathic Medicine, Provo, UT 84606, USA

**Keywords:** quercetin, diabetes, oxidative stress, inflammation, diabetes complications

## Abstract

Diabetes mellitus is a chronic metabolic disorder linked with several microvascular and macrovascular complications and is one of the major causes of morbidity and mortality worldwide. Uncontrolled hyperglycemia is well known to increase oxidative and inflammatory responses. Further, activation of the polyol pathway and protein kinase C (PKC), and formation of advanced glycation end-products (AGEs) and their interaction with AGE receptors (RAGEs) can cause tissue dysfunction and damage, leading to secondary diabetes complications such as diabetic retinopathy, neuropathy, nephropathy, cardiovascular complications, and cataractogenesis. Several plant products have been shown to exert potential glucose-lowering effects due to their strong antioxidant and anti-inflammatory properties. Quercetin, a naturally occurring flavonoid abundant in fruits and vegetables, has attracted considerable interest because of its antioxidant, anti-inflammatory, and cytoprotective properties. Recent studies indicate the therapeutic potential of quercetin for controlling secondary diabetes complications. Various preclinical studies suggest that quercetin prevents the production of reactive oxygen species, suppresses NF-κB-mediated inflammatory signaling, activates Nrf-2-mediated antioxidant enzymes, and inhibits AGE-RAGE interactions. These actions of quercetin have been shown to prevent the production of pro-inflammatory cytokines, enhance endogenous antioxidant defenses, and attenuate the pathological consequences of diabetes complications. Although preclinical findings convincingly support the protective effects of quercetin across multiple diabetic complications, clinical findings remain limited and heterogeneous. Further, randomized clinical trials, long-term safety studies, and bioavailability studies are needed to determine its therapeutic effectiveness in clinical practice. In this review, we discuss recent findings on how quercetin prevents various secondary complications of diabetes. We further discuss how quercetin could serve as a promising multi-target adjunctive therapy by addressing the oxidative and inflammatory mechanisms underlying diabetic complications.

## 1. Introduction

Despite recent progress in medicine, diabetes mellitus (DM) remains one of the most common chronic diseases worldwide, with increasing morbidity and mortality [1]. Specifically, the complications of diabetes are increasing due to poor lifestyle habits, lack of physical activity, and increased consumption of carbohydrate-rich foods [2,3]. Several studies clearly show that uncontrolled blood glucose levels, by activating various oxidative and inflammatory pathways, could lead to secondary diabetes complications [4,5,6]. These complications include diabetic retinopathy, nephropathy, neuropathy, cardiovascular complications, and cataracts. Further, recent epidemiological studies also indicate that in the United States, the diabetes burden has increased significantly from 1990 to 2024 [7]. Specifically, the combined incidence rate of type 1 and type 2 DM increased 15-fold. In contrast, the incidence of type 2 DM alone increased 20-fold, indicating that existing preventive measures and treatment options are insufficient to control disease progression [7].

**Figure 1:**
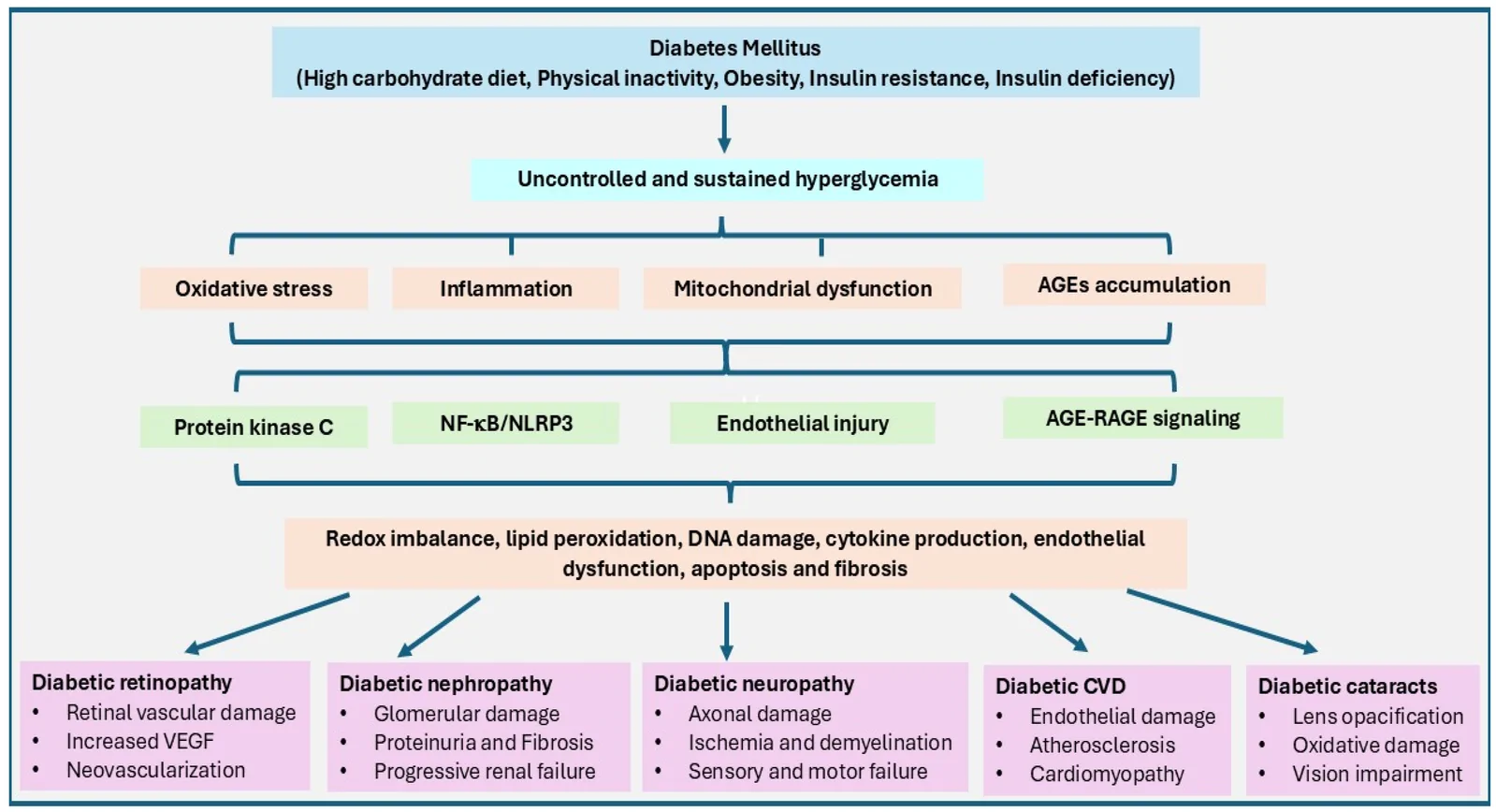
Molecular mechanisms involved in the pathophysiology of secondary diabetic complications. Diabetes mellitus associated with metabolic and lifestyle factors can lead to uncontrolled and sustained hyperglycemia. Chronic hyperglycemia induces oxidative stress, inflammation, mitochondrial dysfunction, and accumulation of advanced glycation end products (AGEs). Hyperglycemia acts through PKC/NF-κB/NLRP3 signaling/AGE–RAGE signaling, leading to progressive tissue injury in secondary diabetic complications like diabetic retinopathy, nephropathy, neuropathy, cardiovascular disease (CVD), and cataracts.

Despite recent medical advances to control blood glucose levels, the secondary diabetic complications continue to affect millions of individuals [8]. Thus, uncontrolled and sustained hyperglycemia is still a leading cause of kidney failure, peripheral neuropathy, cardiovascular disease, and visual impairment in diabetic patients. Recent studies also suggest that prolonged hyperglycemia could initiate various metabolic disturbances such as oxidative stress, chronic inflammation, mitochondrial dysfunction, endothelial injury, and accumulation of advanced glycation end products (AGEs) [9,10]. These events modulate signaling pathways mediated by protein kinase C (PKC), receptors of AGEs (RAGEs), nuclear factor-kappa binding protein (NF-κB), transforming growth factor-β (TGF-β), and vascular endothelial growth factor (VEGF), thereby leading to progressive cellular dysfunction and tissue damage (Figure 1).

Furthermore, growing evidence suggests that oxidative stress represents a common molecular mechanism linking the various complications of diabetes [6,9,11,12]. Hyperglycemia-induced excessive production of reactive oxygen species (ROS) disrupts endogenous antioxidant defenses and mitochondrial function [13]. This redox imbalance could lead to lipid peroxidation, DNA damage, inflammatory cytokine production, endothelial dysfunction, apoptosis, and fibrosis. These processes have been shown to contribute to the progression of diabetic retinopathy, nephropathy, neuropathy, cardiovascular disease, and cataract formation. Indeed, several antioxidants have been shown to prevent secondary diabetic complications by restoring redox homeostasis [11,12,13].

Quercetin (3,5,7,3′,4′-pentahydroxyflavone) is a naturally occurring flavonoid compound commonly found in various plants [14]. Quercetin has strong antioxidative and anti-inflammatory properties [15]. It has been shown to directly scavenge ROS, enhance nuclear factor erythroid 2-related factor 2 (Nrf-2)-dependent antioxidant enzyme activity, suppress NF-κB-dependent pro-inflammatory signaling, and inhibit AGE-RAGE interactions [16]. Numerous preclinical studies have also demonstrated beneficial effects of quercetin in diabetic tissues [17]. Quercetin has been shown to preserve retinal microvasculature [18], attenuate retinal oxidative stress [19], and prevent cataractogenesis [20], thus improving vision problems in diabetic models. Similarly, by protecting neuronal cells from death and reducing cardiovascular remodeling, it could prevent neuropathy and diabetic cardiovascular complications, respectively [21,22]. Although encouraging evidence has emerged from preclinical animal models and selected clinical studies, the translational potential of quercetin in diabetes remains incompletely defined.

This comprehensive narrative review discusses the current understanding of the molecular mechanisms underlying major diabetic complications. We also critically analyze evidence supporting quercetin as a multi-target therapeutic agent for diabetes. We also emphasize that the antioxidant, anti-inflammatory, anti-apoptotic, anti-fibrotic, and anti-glycation activities of quercetin help control diabetic retinopathy, nephropathy, neuropathy, cardiovascular disease, and cataracts. By integrating these findings, this review intends to evaluate quercetin’s potential as an adjunctive strategy to reduce the burden of diabetes-related complications. Further, there are very few reviews in the past 5 years that have discussed quercetin’s therapeutic potential in diabetes. However, this review differs from earlier publications by comprehensively evaluating the molecular mechanisms underlying quercetin’s protective effects across five major secondary diabetic complications: diabetic retinopathy, nephropathy, neuropathy, cardiovascular disease, and cataractogenesis. We have conducted a PubMed search to identify relevant studies published within the last 10 years, with a few exceptions from 2000 to 2015. We included a few of these earlier publications because they provide foundational mechanistic insights and establish key molecular pathways essential to understanding quercetin’s therapeutic potential. Predefined keywords included diabetes mellitus, quercetin, diabetic retinopathy, diabetic neuropathy, diabetic nephropathy, diabetic cardiovascular complications, diabetic cataracts, and their combinations. We included original preclinical research articles, clinical studies, and various reviews, including narrative reviews, systematic reviews, and meta-analyses. We excluded studies showing quercetin involvement in non-diabetes-related diseases such as inflammatory complications, infections, and cancer.

## 2. Quercetin in Diabetic Retinopathy

Diabetic retinopathy (DR) is a multi-stage disease complication that commonly results from poorly controlled DM [22]. It mainly results from damage and abnormal changes to the blood vessels near the retina, the posterior region of the eye that contains the photoreceptors responsible for vision [23]. These vascular changes could lead to impaired vision and, in more severe cases, complete vision loss. DR is a major cause of blindness in the United States, and it was estimated that in 2021, 9.60 million people were living with this disease, corresponding to 26% of people with DM [24]. Simó-Servat et al. [25] have indicated that high hemoglobin (Hb)A1C and the presence of hypertension are among the most relevant risk factors for the development and progression of DR.

DR can present as an early non-proliferative stage that can progress to a more serious proliferative stage. Non-proliferative DR appears as changes in the retinal vasculature. These changes can include capillary swelling and rupture, leading to exudate accumulation and vision loss [26,27]. Proliferative DR involves the development of new, abnormal vasculatures along the retina and optic disk [28]. Further, these vascular changes have been linked to increased inflammation and oxidative stress caused by poorly controlled hyperglycemia. For example, Yue et al. [29] showed that oxidative stress generates reactive oxygen species (ROS) that can alter biochemical pathways, leading to the signs and symptoms associated with DR.

Furthermore, prolonged hyperglycemia has been shown to regulate multiple oxidative pathways, causing microvascular damage and uncontrolled retinal vasculature growth [30]. Hyperglycemia can also increase the levels of AGEs. Higher AGE levels, by interacting with the RAGE, activate signaling pathways associated with oxidative stress and inflammation [31,32]. Khalid et al. [33] have shown that hyperglycemia-associated AGEs and ROS cause retinal damage and dysfunction in DR.

Another significant downstream effect of increased AGE levels is increased RAGE receptor activation. This receptor is closely linked to ROS production as well as inflammatory signaling within the retinal vasculature [34,35]. These changes can increase capillary permeability and contribute to VEGF signaling pathway activation [36]. VEGF pathway activation can lead to vascular leakage and impaired vision. Upregulation of VEGF can also lead to abnormal angiogenesis, a key feature of proliferative DR [36,37]. This unregulated growth of new vasculature can cause blood vessels to encroach on photosensitive areas of the eye, impairing vision.

Current treatments for DR are limited. In the early stages of this disease, early detection and proper blood glucose control are among the most common treatment recommendations. In more severe cases, administration of anti-VEGF injections could reduce the growth of new, abnormal blood vessels [38]. Still, this treatment targets only one of the many pathways causing the devastating effects of DR. Surgical procedures such as vitrectomy and laser photocoagulation may also be used to manage advanced DR and its complications [39].

**Figure 2:**
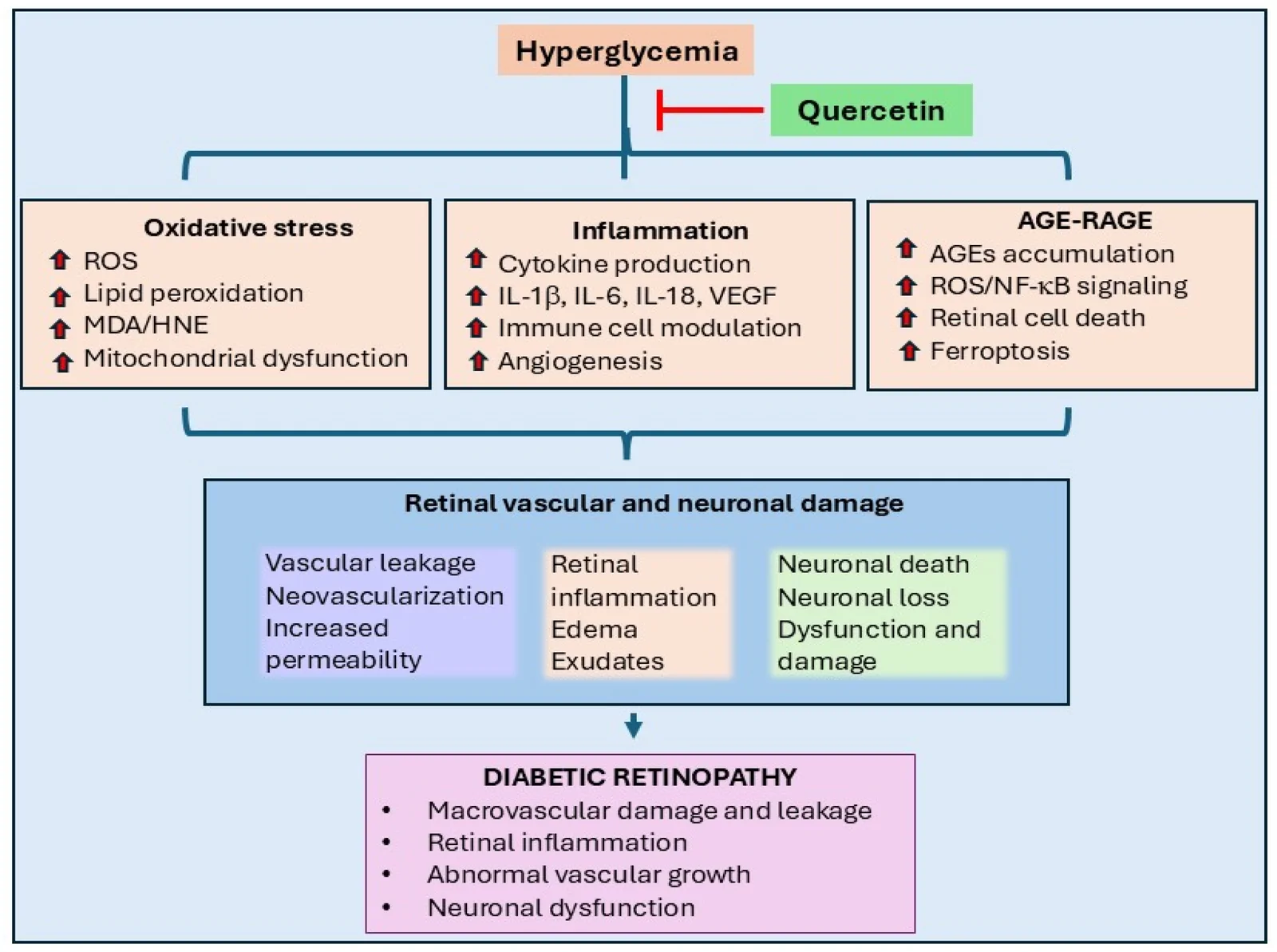
Protective effects of quercetin against diabetic retinopathy. Sustained hyperglycemia leads to retinal vascular leakage, neovascularization, edema, and neuronal injury, leading to diabetic retinopathy. Quercetin may attenuate the progression of diabetic retinopathy by targeting hyperglycemia-induced oxidative, inflammatory, and AGE–RAGE pathways, limiting retinal vascular and neuronal damage.

A few preclinical studies show the significance of quercetin in DR. These studies indicate that quercetin, by inhibiting pro-inflammatory pathways and activating antioxidative pathways, prevents retinal damage and dysfunction in preclinical animal models (Figure 2).

Ola et al. [40] showed that quercetin treatment in diabetic rats increased neurotrophic factors such as brain-derived neurotrophic factor (BDNF), nerve growth factor (NGF), and synaptophysin, and increased the anti-apoptotic protein B-cell lymphoma-2 (Bcl-2), while reducing cytochrome c release and caspase-3 activity in the retina, suggesting that quercetin may prevent neuronal apoptosis. Another study by Lee et al. [41] showed that quercetin (0.1 μM) prevents VEGF-induced inflammatory responses in mouse retinal photoreceptors. Specifically, they showed that quercetin restored the VEGF-induced decrease in zonula occludens-1 and β-catenin expression and suppressed VEGF-induced protein kinase B (AKT), mitogen-activated protein kinase (MAPK), and NF-κB signaling in these cells. Similarly, Chen et al. [42] showed that quercetin decreased matrix metalloproteinase 9 (MMP9) and VEGF levels in retinal tissues from a diabetic rat model of DR. They also indicated that quercetin reduced retinal edema and vacuolar pathological changes.

In another study, Lupo et al. [43] showed that, compared with quercetin (25 μM), its derivative 8-methylquercetin (25 μM) pentamethyl ether more robustly prevented VEGF-A-induced AKT, extracellular signal-regulated kinase (ERK), and c-Jun N-terminal kinase (JNK) activation in human microvascular endothelial cells. They also showed that these compounds prevented migration and microvessel formation in rabbit aortic rings, suggesting that quercetin and its derivatives may inhibit angiogenesis, an important contributor to DR. Interestingly, in a zebrafish model of DR, a quercetin nano-formulation (5 and 10 mg/kg) attenuated DR features by reducing homocysteine levels and lipid peroxidation and increasing free-radical scavenging activity [44].

A preclinical study by Chai et al. [45] showed that quercetin (150 mg/kg/day) reduced the expression of interleukin (IL)-1β, IL-18, IL-6, and tumor necrosis factor-alpha (TNF-α) in the retinal tissues of a streptozotocin (STZ)-induced rat type 1 DR model. They also showed that quercetin inhibited NF-κB signaling and NLR family pyrin domain containing 3 (NLRP3) inflammasome activation and increased heme oxygenase-1 (HO-1) expression. This study indicated that quercetin may protect against DR by decreasing inflammatory responses and increasing antioxidant responses. Similarly, Li et al. [46] showed that in human retinal microvascular endothelial cells, quercetin (20, 40 and 80 μM) inhibited high glucose-induced NLRP3, apoptosis-associated speck-like protein containing a CARD (ASC), and caspase-1 activation and reduced the release of IL-1β and IL-18. The data also showed that quercetin inhibited high glucose-induced autophagy in these cells. On the other hand, Liu et al. [47] showed that quercetin (5 or 20 μM) reduced hyperglycemia-induced VEGF-A expression by inhibiting the ROS/p38 MAPK/NF-κB signaling pathway in retinal pigment epithelial cells. In addition, Cai et al. [48] showed that isoquercetin, a water-soluble form of quercetin, attenuated DR in a mouse model. Specifically, they showed that isoquercetin reduced p53-mediated ferroptosis and retinal damage.

Flavonoids like quercetin show therapeutic potential by reducing ROS and overall inflammation [49,50]. Malondialdehyde (MDA) and 4-hydroxynonenal (4-HNE) are toxic byproducts of free-radical-mediated lipid oxidation [51]. Higher levels of these lipid aldehydes demonstrate increased oxidative stress within a system [52]. A few studies indicate increased levels of these aldehydes in patients with DR [53,54,55] and quercetin has been shown to reduce the formation of these aldehydes, indicating that quercetin, by reducing oxidative stress, could improve DR [56]. Together with experimental studies demonstrating quercetin’s antioxidant and anti-inflammatory effects in retinal cells and animal models of DR, the results support further investigation of quercetin in clinical settings as a possible therapeutic approach for DR.

Thus, based on the current evidence, DR is still one of the most common complications resulting from DM and is a growing concern globally. Hyperglycemia-associated inflammation and oxidative stress could damage the retina’s delicate microvasculature and neural components, leading to DR [57,58]. Recent studies indicate that quercetin may target several pathways involved in DR, including oxidative stress, inflammation, VEGF signaling, angiogenesis, neuronal apoptosis, and ferroptosis [56]. However, much of the current evidence comes from cell culture and animal studies, and additional clinical studies are needed to determine whether quercetin can safely and effectively prevent or treat DR in humans.

## 3. Quercetin in Diabetic Nephropathy

Diabetic nephropathy (DN) is a microvascular complication that leads to end-stage renal disease (ESRD) [59]. DN is characterized mainly by persistent albuminuria and reduced estimated glomerular filtration rate (eGFR). Early pathological changes also include glomerular hyperfiltration, glomerular basement membrane thickening, and mesangial expansion [60]. Specifically, individuals with severely increased albuminuria (≥300 mg/g) are at greater risk of progressive decline in kidney function leading to nephropathy [61].

In addition to chronic hyperglycemia, hypertension, obesity, oxidative stress, and inflammation contribute to DN development through several pathways [62]. Several studies have shown that hyperglycemia promotes reactive oxygen species (ROS) production and activates inflammatory mediators, including TNF-α, IL-6, transforming growth factor-beta (TGF-β), and intercellular adhesion molecule-1 (ICAM-1) [63,64,65]. These changes are associated with NF-κB activation and increased inflammatory signaling in renal tissue [66]. Hyperglycemia also increases TGF-β signaling and contributes to renin–angiotensin system activation [67]. Angiotensin II increases glomerular capillary pressure, whereas TGF-β promotes extracellular matrix accumulation, mesangial expansion, and renal fibrosis [68]. Further, AGEs have also been shown to contribute to renal injury by interacting with RAGE. The AGE/RAGE axis promotes oxidative stress and inflammatory responses, leading to renal cell damage and dysfunction [69,70].

**Figure 3:**
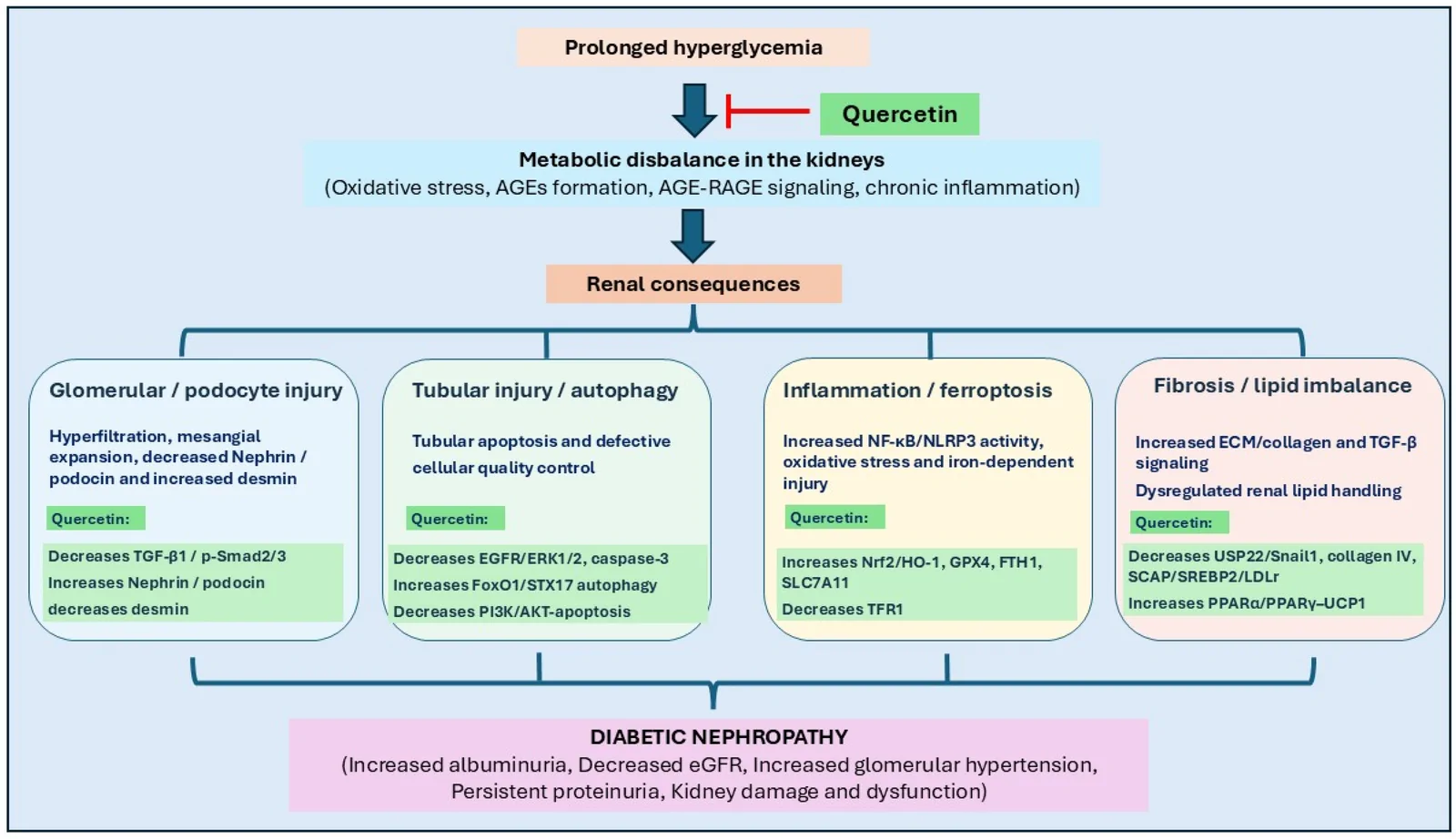
Protective effects of quercetin against diabetic nephropathy. Prolonged hyperglycemia induces metabolic imbalance in the kidneys and promotes glomerular alterations, podocyte injury, tubulointerstitial inflammation, and extracellular matrix (ECM) accumulation and fibrosis through nephrin and podocin loss. Quercetin may attenuate diabetic nephropathy by targeting hyperglycemia-associated oxidative, inflammatory, and profibrotic pathways, limiting renal cellular and structural injury.

Current management of DN includes glycemic and blood pressure control, angiotensin-converting enzyme inhibitors (ACEi) or angiotensin receptor blockers (ARBs), and sodium-glucose cotransporter-2 (SGLT2) inhibitors [71]. Although these treatments can slow disease progression, DM patients may continue to experience albuminuria and progressive loss of kidney function [72]. Because oxidative stress, inflammation, fibrosis, and renal cell injury contribute to DN, compounds that affect several of these processes have received considerable attention [73,74]. Quercetin has been investigated for its antioxidant, anti-inflammatory, anti-fibrotic, and renal protective properties in DN [75,76,77]. These studies indicate potential beneficial effects of quercetin in controlling the development of DN (Figure 3).

Several animal studies have examined quercetin’s effects on renal injury in DN. Tong et al. [77] showed that diabetic mice developed increased glomerular volume, renal cortical inflammatory cell infiltration, basement membrane changes, oxidative stress, and kidney injury. Quercetin (10 mg/kg) treatment reduced these histological changes and decreased oxidative and inflammatory injury. Similarly, Abdou et al. [78] showed that quercetin (50 mg/kg) treatment in streptozotocin (STZ)-induced type 1 diabetic rats reduced renal biochemical abnormalities, oxidative stress, and inflammatory mediators, including TGF-β, TNF-α, IL-6, and glycogen synthase kinase-3 beta (GSK-3β). Further, they showed that quercetin also increased antioxidant defenses, including glutathione (GSH), catalase, and total antioxidant capacity. Hossein et al. [79] further showed that quercetin (50 mg/kg) reduced renal dysfunction, lipid peroxidation, oxidative stress, and histological damage in STZ-induced type 1 diabetic rats. These effects were associated with changes in apelin/TGF-β signaling, and quercetin pretreatment produced greater improvement than treatment initiated after diabetes induction.

Quercetin has also been shown to affect inflammatory cell responses. For example, Abudoureyimu et al. [80] showed that quercetin reduced M1 macrophage polarization and decreased TNF-α, IL-6, and IL-1β in high glucose-treated macrophages. Quercetin (50 and 100 mg/kg) also increased markers associated with M2 macrophages and reduced renal inflammation and fibrosis in diabetic mice. These effects were linked to the inhibition of the NLRC5/NLRP3 pathway, and NLRC5 overexpression reduced quercetin’s anti-inflammatory effects. Thus, regulating macrophage responses may contribute to quercetin’s effects on renal inflammation in experimental DN.

Podocyte injury is an important pathological feature of DN and contributes to albuminuria. Gao et al. [81] showed that quercetin (50 and 100 mg/kg/day) treatment for 12 weeks reduced albuminuria, renal dysfunction, oxidative stress, and structural kidney damage in STZ-induced type 1 diabetic rats. Quercetin increased the expression of the podocyte proteins nephrin and podocin and decreased the podocyte injury marker desmin. Further, they showed that quercetin reduced TGF-β1, phosphorylated Smad2, and phosphorylated Smad3 and increased Smad7, indicating inhibition of TGF-β1/Smad signaling. These results indicate that quercetin reduces podocyte injury by limiting oxidative stress and TGF-β1/Smad signaling. Similarly, Liu et al. [82] examined podocyte apoptosis in db/db mice and high glucose-treated mouse podocytes. Quercetin (50–150 mg/kg/day) decreased the pro-apoptotic proteins Bax and cleaved caspase-3 and increased the anti-apoptotic protein Bcl-2. Quercetin also reduced EGFR and ERK1/2 phosphorylation. These findings indicate that inhibition of EGFR/ERK1/2 signaling is associated with quercetin’s anti-apoptotic effects in podocytes. More recently, Hou et al. [83] showed that quercetin (50 mg/kg/day) restored autophagic flux in a mouse model of DN without substantially altering upstream autophagy initiation. Further, quercetin has been shown to regulate FoxO1 and promote STX17-dependent autophagosome-lysosome fusion [83]. Mesangial cell proliferation and dysfunction could also contribute to glomerular changes in DN. Lei et al. [84] showed that high glucose induced proliferation of mouse glomerular mesangial cells and suppressed Hippo signaling, increasing YAP nuclear translocation and YAP/TEAD-mediated expression of proliferative genes. Quercetin reactivated Hippo signaling, reduced YAP activity, and inhibited mesangial cell proliferation. Similar effects were observed in db/db mice, in which quercetin reduced mesangial cell proliferation and improved renal function [84].

Similarly, Wan et al. [85] also examined the effects of quercetin on high glucose-treated human mesangial cells. Quercetin reduced cell proliferation, inflammation, and oxidative stress. This study showed that miR-485-5p directly targeted Yes-associated protein 1 (YAP1) and that quercetin regulated the miR-485-5p/YAP1 axis. Serum from patients with DN also showed reduced miR-485-5p and increased YAP1. Similarly, Xu et al. [86] showed that glucose fluctuations increased inflammation, apoptosis, and aerobic glycolysis, leading to mesangial cell dysfunction. Quercetin reduced these changes and regulated the HIF-1α/miR-210/ISCU/FeS pathway while preserving Fe-S-dependent mitochondrial enzyme function. These studies indicate that quercetin affects both proliferative signaling and metabolic abnormalities in mesangial cells exposed to diabetic conditions.

Renal fibrosis is another major pathological component of progressive DN. Zhao et al. [87] showed that quercetin (20 and 40 μM) reduced epithelial-to-mesenchymal transition (EMT), extracellular matrix accumulation, and tubulointerstitial fibrosis in high glucose-treated renal tubular epithelial cells and diabetic mice. Quercetin inhibited ubiquitin-specific peptidase 22 (USP22) and reduced USP22-mediated deubiquitination and stabilization of Snail1, leading to decreased accumulation of fibronectin and collagen IV. This study also showed that in diabetic mice, quercetin also decreased renal USP22 and Snail1 levels and reduced tubulointerstitial fibrosis. Another study examined the relationship between quercetin, renal fibrosis, and sirtuin 5 (SIRT5) levels [88]. STZ-induced type 1 model of diabetic rats showed impaired renal function, increased albuminuria, glycogen deposition, collagen accumulation, and increased renal SIRT5 expression. Quercetin treatment (30, 60, and 120 mg/kg) improved renal function and pathological changes and reduced SIRT5 protein levels. Although these observations associate SIRT5 with quercetin’s effects, additional studies are needed to determine whether changes in SIRT5 directly mediate its anti-fibrotic effects.

Ferroptosis has recently been examined as another mechanism of renal cell injury in DN. Feng et al. [89] showed that high glucose induced ferroptosis in renal tubular epithelial cells. Quercetin (6.25 to 100 μM) decreased transferrin receptor 1 (TFR1) and increased glutathione peroxidase 4 (GPX4), FTH1, and SLC7A11 in high glucose-treated HK-2 cells. Quercetin also increased nuclear factor erythroid 2-related factor 2 (Nrf2) and heme oxygenase-1 (HO-1), linking its anti-ferroptotic effects to the activation of the Nrf2/HO-1 pathway. Similarly, Zhang et al. [90] showed that quercetin reduced ferroptosis-associated renal tubular injury in a diabetic kidney disease rat model and high glucose-treated HK-2 cells. Increased Nrf2 expression was associated with quercetin’s protective effects, further supporting a link between Nrf2 activation and suppression of tubular ferroptosis. Furthermore, Wu et al. [91] also investigated quercetin-4’-O-β-D-glucopyranoside (QODG), a quercetin derivative, in high glucose-treated podocytes and diabetic mice. QODG (50, 100, and 150 µM) reduced oxidative stress, renal injury, and ferroptosis. Because this study examined a quercetin derivative rather than quercetin itself, these findings support the activity of a structurally related flavonoid but should be distinguished from studies using quercetin.

Another study by Liu et al. [92] showed that quercetin (150 mg/kg/day) reduced renal tubular epithelial cell apoptosis in high-fat diet and STZ-induced type-2 diabetic mice and high glucose-treated human kidney-2 (HK-2) cells. Quercetin inhibited high glucose-induced PI3K/AKT signaling, and this effect was associated with reduced tubular epithelial cell apoptosis and improvement in renal injury. Thus, quercetin may affect several forms of renal cell injury, including apoptosis and ferroptosis.

Abnormal renal lipid accumulation and lipotoxicity can contribute to kidney injury in diabetes. For example, Jiang et al. [93] showed that quercetin (50 or 100 mg/kg) reduced albuminuria, renal dysfunction, and lipid accumulation in db/db mice while improving glucose and lipid metabolism. Quercetin decreased low-density lipoprotein receptor (LDLr), HMG-CoA reductase (HMGCR), sterol regulatory element-binding protein-2 (SREBP-2), and SREBP cleavage-activating protein (SCAP) expression, indicating regulation of the SCAP/SREBP2/LDLr pathway. More recently, Yong et al. [94] showed that quercetin reduced renal lipid accumulation, fibrosis, and lipotoxicity-associated injury in experimental diabetic kidney disease. They identified peroxisome proliferator-activated receptor (PPAR)α and PPARγ as targets involved in these effects. Quercetin activated the PPARα/PPARγ-uncoupling protein 1 axis, increased fatty acid oxidation, and reduced ROS production.

Furthermore, a few studies also indicate that quercetin’s relatively low bioavailability may limit its therapeutic use in DN. For example, Tang et al. [95] compared free quercetin with pegylated quercetin liposomes in STZ-induced diabetic rats. Both preparations improved renal biochemical parameters, oxidative stress, and pathological changes, but pegylated quercetin liposomes (200 mg/kg/day) produced greater effects and maintained higher plasma quercetin concentrations than free quercetin (50 mg/kg/day). These findings suggest that modifying quercetin delivery may increase its systemic exposure and renal effects. Similarly, a few studies also investigated the significance of quercetin in combination with other pharmacological agents. Zhu et al. [96] showed that dasatinib plus quercetin improved renal function, albuminuria, and kidney histopathology in db/db mice while reducing extracellular matrix accumulation. The combination increased autophagy and preserved podocyte differentiation, with the Notch pathway implicated in these effects. Further, Bian et al. [97] also showed that short-term dasatinib plus quercetin treatment reduced glomerular and tubular injury, fibrosis, cellular senescence, and inflammation in diabetic mice. Treatment increased α-Klotho and Sirtuin-1 and reduced NF-κB-associated inflammation and senescence. On the other hand, Guo et al. [98] further reported that dasatinib plus quercetin improved renal function, fibrosis, lipid accumulation, and cellular energy metabolism through effects associated with PPARα and fatty acid oxidation. Because these studies administered dasatinib and quercetin together, the renal effects cannot be assigned specifically to quercetin. Additionally, in rat kidney epithelial cells, quercetin and atorvastatin reduced TGF-β, TNF-α, and IL-6 and showed antioxidant effects [99]. Combined treatment improved cell viability, although the study was conducted in vitro. Similarly, Gan et al. [100] examined quercetin and rosuvastatin in high glucose-treated renal tubular epithelial cells. Both compounds reduced inflammatory and ferroptosis-associated changes, and combined treatment produced greater effects than either compound alone.

Only a few studies have reported the significance of quercetin in DN in clinical trials. For example, Hickson et al. [101], in a Phase 1 pilot study involving nine patients with diabetic kidney disease, examined a three-day course of dasatinib (100 mg) plus quercetin (1000 mg). Treatment reduced markers of cellular senescence in adipose tissue and skin and decreased macrophage accumulation and circulating senescence-associated secretory phenotype factors, including IL-1α, IL-6, MMP-9, and MMP-12. This study demonstrated biological effects of the senolytic combination in humans but was small, uncontrolled, and did not establish improvement in kidney function. In addition, the use of dasatinib with quercetin prevents attribution of these effects to quercetin alone. In addition, Liu et al. [102] examined the association between dietary quercetin intake and DN using National Health and Nutrition Examination Survey (NHANES) data from 2279 adults with diabetes. They found that higher dietary quercetin intake was associated with lower odds of DN after adjustment for potential confounders. The results suggest that a 1-unit increase in log-transformed dietary quercetin intake was associated with approximately 38% lower odds of DN. Although this study provides human observational evidence, it does not demonstrate that quercetin intake can prevent DN or improve established kidney disease.

A few systematic reviews and meta-analyses have evaluated the experimental evidence of quercetin in controlling DN. For example, Feng et al. [103] analyzed 20 preclinical studies involving 378 animals and reported improvements in several renal function indices following quercetin treatment. Quercetin decreased MDA and inflammatory mediators, including TNF-α and IL-1β, while increasing SOD and catalase activities. However, creatinine clearance did not improve significantly. Similarly, Hu et al. [104], in a systematic review and meta-analysis of 20 animal studies, reported that quercetin decreased serum creatinine, BUN, urinary protein, urinary albumin, MDA, TNF-α, and IL-1β and increased superoxide dismutase (SOD) and catalase activities. However, creatinine clearance and uric acid levels were not significantly affected. Further, a study by Li et al. [105] analyzed 18 preclinical studies and reported that quercetin improved renal function, glucose and lipid metabolism, oxidative stress, inflammation, fibrosis, and renal histopathology in experimental DN. The proposed mechanisms included TGF-β1/Smad, Nrf2/HO-1, Hippo, PI3K/AKT, SCAP/SREBP2/LDLr, and NLRP3-associated signaling. Although animal models reported dose- and duration-related effects, these findings cannot establish an effective or safe dose of quercetin for patients with DN. Another study by Liu et al. [106] also demonstrated growing interest in quercetin and kidney disease in a bibliometric analysis. The analysis included 1253 publications from 2000 to 2022 and identified oxidative stress and antioxidant activity of quercetin in the prevention of DN.

Thus, current studies show that quercetin affects several pathological processes involved in experimental DN. These include oxidative stress and inflammation, podocyte injury and apoptosis, mesangial cell proliferation, renal fibrosis, tubular epithelial cell injury, ferroptosis, altered autophagy, and renal lipid accumulation. Additional clinical trials are needed to determine whether quercetin alone improves albuminuria, eGFR, or other clinically meaningful renal outcomes and to establish an appropriate dose, formulation, and safety profile for patients with DN.

## 4. Quercetin and Diabetic Neuropathy

Diabetic neuropathy progressively damages peripheral nerves and impairs Schwann cell function [107]. Schwann cells are essential for producing and maintaining myelin, which permits efficient nerve signal conduction [107,108]. Schwann cell dysfunction and myelin sheath deterioration could impair nerve conduction and contribute to sensory and motor abnormalities, including numbness, tingling, pain, and weakness [108,109]. Diabetic neuropathy has been shown to affect approximately 50–60% of patients with diabetes, and its prevalence increases with disease duration and poor glycemic control [110]. Diabetic neuropathy can broadly present as diabetic peripheral neuropathy (DPN) and painful diabetic neuropathy (pDN), with approximately 20–50% of diabetic individuals experiencing painful neuropathy [110,111].

Chronic hyperglycemia is a major contributor to the pathogenesis of diabetic neuropathy by promoting oxidative stress and inflammation. Elevated ROS production causes mitochondrial damage and impairs oxidative phosphorylation, which can damage neuronal membranes and myelin, impair Schwann cell function, and ultimately promote peripheral nerve demyelination [112,113].

Hyperglycemia also increases AGE accumulation in neuronal tissues, including peripheral nerves [114]. AGEs interact with RAGE and stimulate intracellular signaling pathways leading to the production of pro-inflammatory cytokines. Further, RAGE signaling can also activate NADPH oxidase, further increasing ROS production and oxidative damage to lipids, proteins, and DNA [115]. Consequently, these processes damage peripheral nerve tissues and intensify inflammation and nerve injury [115,116,117]. In addition, AGE–RAGE signaling has also been shown to increase the expression of adhesion molecules such as vascular cell adhesion molecule-1 (VCAM-1), facilitating immune-cell adhesion to vascular endothelial cells and contributing to increased vascular permeability and peripheral nerve inflammation [118].

**Figure 4:**
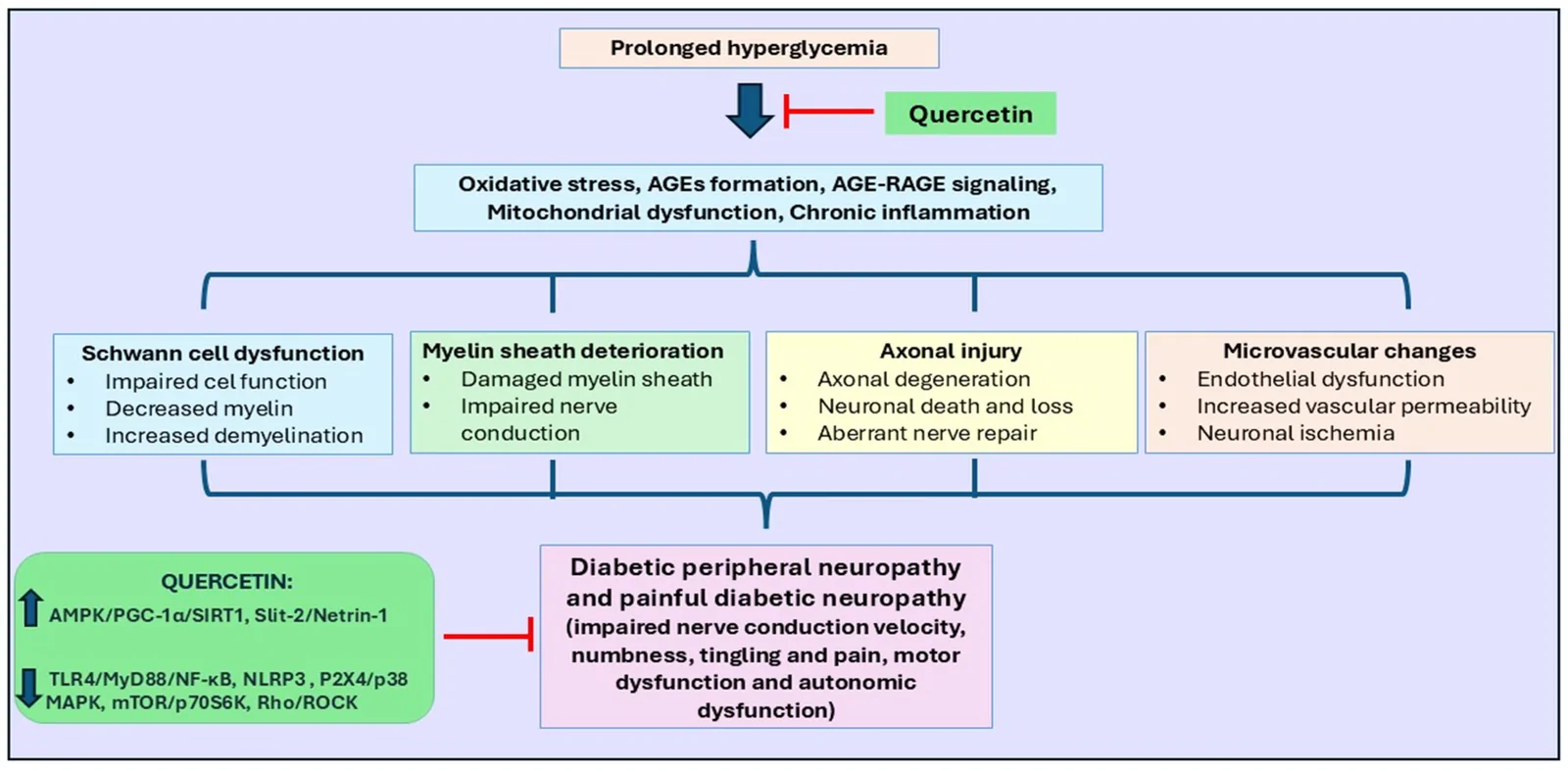
Significance of quercetin in preventing diabetic neuropathy. Prolonged hyperglycemia through increased oxidative stress, AGE formation and AGE–RAGE signaling, and chronic inflammation causes Schwann cell dysfunction, myelin sheath deterioration, axonal injury, and microvascular abnormalities. These pathological changes lead to demyelination, impaired nerve conduction, neuronal ischemia and loss, and defective nerve repair, resulting in sensory symptoms and motor and autonomic dysfunction in diabetic peripheral neuropathy and painful diabetic neuropathy. Quercetin has been shown to attenuate diabetic neuropathy by inhibiting hyperglycemia-associated oxidative, inflammatory, metabolic, and mitochondrial pathways.

Recent studies have shown that flavonoids such as quercetin are potential neuroprotective compounds due to their influence on oxidative stress, inflammatory signaling, mitochondrial function, neuronal survival, myelination, axonal repair, and neuropathic pain [119,120] (Figure 4). The neuroprotective effects of quercetin were reported by de Souza et al. [121]. In diabetic rats, quercetin supplementation (40 mg/kg/day) reduced the loss of jejunal neurons and glial cells, with 23.06% higher neuronal density and 14.55% higher glial cell density compared with untreated diabetic rats. These results indicate that quercetin provides a modest neuroprotective effect against diabetes-associated damage to the enteric nervous system.

Similarly, quercetin’s antioxidant properties have been shown to contribute substantially to its protective effects. For example, Dureshahwar et al. [122] investigated an ethyl acetate fraction of *Allium cepa* leaves containing 4.82% quercetin in STZ-induced diabetic neuropathy. Treatment with the *A. cepa* extract or quercetin (40 mg/kg) improved thermal hyperalgesia, cold allodynia, motor coordination, and biochemical parameters, suggesting that quercetin-rich *A. cepa* extracts may provide neuroprotection through antioxidant and glucose-lowering activities. Similarly, Chis et al. [123] demonstrated that quercetin (30 mg/kg/day) combined with moderate exercise for five weeks reduced diabetes-induced histological alterations in the sciatic nerve and indicated synergistic neuroprotective effects of quercetin.

Beyond reducing oxidative stress, quercetin may protect peripheral nerves by preserving mitochondrial function. For example, Zhang et al. [124] showed that quercetin (30 and 60 mg/kg/day) increased paw withdrawal thresholds and nerve conduction velocity and reduced pathological changes in the sciatic nerve and dorsal root ganglia of STZ-induced type 1 diabetic model rats. Quercetin also reduced oxidative stress, restored ATP production and mitochondrial structure, and increased myelin protein expression. These effects were associated with activation of the AMPK/PGC-1α/SIRT1 pathway, suggesting that improved mitochondrial function and cellular energy metabolism are important mechanisms underlying quercetin’s neuroprotective effects. Similarly, quercetin also appears to exert substantial anti-inflammatory activity in diabetic peripheral nerves. Zhao et al. [125] showed that quercetin (30 and 60 mg/kg/day) improved mechanical withdrawal thresholds, nerve conduction velocity, and sciatic nerve pathology in type 1 diabetic model rats without significantly influencing blood glucose levels or body weight. The results also suggest that quercetin may protect peripheral nerves by inhibiting the TLR4/MyD88/NF-κB signaling pathway. More recently, Chen et al. [126] showed that quercetin (25, 50 and 100 mg/kg/day) improved pain thresholds, sciatic nerve morphology, and remyelination-related gene expression in STZ-induced diabetic peripheral neuropathy without substantially altering blood glucose or body weight within 12 weeks. Quercetin also reduced oxidative stress and neuroinflammation in diabetic rats and high glucose-exposed Schwann cells. These effects have been shown to involve suppression of the TXNIP/NLRP3 inflammasome signaling pathway [126].

Beyond its antioxidant and anti-inflammatory actions, quercetin could also directly influence molecular pathways involved in diabetic neuropathic pain. A study by Yang et al. [127] has demonstrated that quercetin reduced neuropathic pain in a type 2 diabetic rat model within 14 days. Quercetin (50 mg/kg/day) suppressed diabetes-induced upregulation of P2X4 receptors in dorsal root ganglion satellite glial cells and reduced downstream p38MAPK activation, indicating that quercetin alleviates mechanical and thermal hyperalgesia by inhibiting P2X4 receptor-mediated p38MAPK signaling in the dorsal root ganglia. Further, Wang et al. [128] showed that chronic quercetin (50 and 100 mg/kg/day) treatment decreased thermal hyperalgesia and reversed diabetes-associated alterations in spinal dorsal horn neurons of db/db mice. Quercetin decreased dendritic branching and spine density and reduced the elevated expression of the synaptic plasticity-associated proteins PSD-95 and synaptophysin. These effects were accompanied by the inhibition of the mTOR/p70S6K signaling pathway, suggesting that quercetin may alleviate diabetic neuropathic pain by limiting maladaptive synaptic plasticity within spinal dorsal horn neurons.

Gut microbiota modulation could provide another mechanism connecting quercetin with oxidative stress and neuropathic phenotypes. For example, Xie et al. [129] showed that quercetin improved mechanical withdrawal thresholds, intraepidermal nerve fiber density, sciatic nerve structure, and neurological markers in STZ-induced diabetic rats. Quercetin also reduced oxidative stress and partially corrected gut dysbiosis by decreasing potentially pathogenic species and enriching beneficial species associated with DPN phenotypes and ROS levels. These data suggest that quercetin’s neuroprotective activity may involve gut microbiota-modulating effects. In addition to preventing neuronal injury, quercetin may promote recovery by promoting axonal protection and nerve repair. Song et al. [130] showed that quercetin (30 and 60 mg/kg/day) improved mechanical and thermal responses, motor nerve conduction velocity, and sciatic nerve pathology in STZ-induced diabetic peripheral neuropathy. The treatment increased the expression of the axonal growth and guidance factors Slit-2 and Netrin-1 while decreasing abnormal activation of the Rho/ROCK signaling pathway. This may contribute to quercetin-mediated axonal protection and peripheral nerve regeneration. Similarly, Thipkaew et al. [131] provided additional evidence for functional nerve recovery by developing quercetin-loaded zein nanofibers and evaluating their effects on sciatic nerve recovery in STZ-induced diabetic rats with peripheral neuropathy. Quercetin-loaded nanofibers have also been shown to improve functional recovery, including walking performance, reflex responses, and nerve conduction, by modulating the pERK/ERK signaling pathway at the nerve injury site.

A few combination studies also suggest that quercetin’s neuroprotective activity may be enhanced when administered with other bioactive compounds. Shi et al. [132] showed that high glucose increased oxidative stress and apoptosis in dorsal root ganglion neurons by increasing ROS production and activating NF-κB signaling. Treatment with quercetin, cinnamaldehyde, and hirudin (QCH) reduced ROS production, caspase-3 activation, IL-6, TNF-α, and NF-κB signaling while increasing the antioxidant Nrf2/HO-1 pathway. Importantly, the three-compound QCH combination provided greater neuroprotection than individual compounds or two-compound combinations, showing a synergistic protective effect. Consistent with these data, Liu et al. [133] demonstrated that high glucose impaired Schwann cell differentiation and myelination, whereas treatment with quercetin, hirudin, and cinnamaldehyde (QHC) promoted Schwann cell differentiation, ciliary neurotrophic factor secretion, and expression of myelin-related proteins. QHC increased both the number and length of myelin segments and produced greater effects than individual compounds or two-compound combinations. The protective effects were associated with the inhibition of the ERK signaling pathway, suggesting that the combination may facilitate Schwann cell differentiation and remyelination in diabetic neuropathy. Similarly, Turedi et al. [134] evaluated quercetin and propolis, individually and in combination, in STZ-induced diabetic rats. Treatment reduced hyperglycemia, oxidative stress, apoptosis, TNF-α, and IL-1β while improving sciatic nerve axonal and myelin morphology. The combined propolis–quercetin treatment provided substantial morphological and neuroprotective effects in diabetic neuropathy when compared to quercetin treatment alone. Further, Harangus et al. [135] evaluated quercetin, pioglitazone, insulin, and a novel thiazolidine-2,4-dione derivative (TZDd) in an STZ-induced type 1 diabetic peripheral neuropathy model. All treatments improved sensory and motor nerve conduction and reduced mechanical hyperalgesia, supporting quercetin’s neuroprotective and functional effects in DPN. To show that novel drug-delivery approaches may further enhance quercetin’s therapeutic potential, Usman Abid et al. [136] developed a nanoethosomal keratolytic gel containing a quercetin–boronic acid complex, epalrestat, and urea to improve transdermal drug delivery for diabetic neuropathic pain. This formulation also enhanced epalrestat skin permeation, and quercetin-containing nanoethosomes exhibited greater antioxidant effects than quercetin alone. In diabetic rats, the formulation reduced neuropathic pain threefold.

Collectively, these preclinical studies show that quercetin acts on multiple pathological processes involved in diabetic neuropathy rather than through a single molecular target. Its reported effects include suppression of oxidative stress and neuroinflammation, preservation of mitochondrial function, modulation of pain signaling and synaptic plasticity, protection of Schwann cells and myelin, promotion of axonal repair, and modulation of the gut microbiota. However, because much of the current evidence is derived from cellular and animal models, further studies are needed to determine whether these effects translate into clinically meaningful benefits in patients with diabetic peripheral neuropathy.

## 5. Quercetin in Diabetic Cardiovascular Diseases

Cardiovascular diseases (CVDs) are the leading cause of death worldwide and among patients with diabetes [137,138]. CVDs encompass disorders affecting the heart and blood vessels. The vasculature transports oxygen and nutrients to major organs, including the heart, and damage to these vessels can alter hemodynamics and increase cardiac stress. Major physiological risk factors include hyperglycemia, hypertension, and elevated cholesterol. Persistent abnormalities in these factors are able to progressively damage blood vessels and increase the risk of coronary artery disease (CAD), myocardial infarction, heart failure, and stroke [139,140].

Recent studies clearly showed that diabetes is strongly associated with the risk of atherosclerosis and other cardiovascular complications [138,139,140]. Chronic hyperglycemia has been shown to promote inflammation, oxidative stress, and vascular endothelial dysfunction, causing vessel stiffening, narrowing, and plaque formation [141]. Rask-Madsen and King [142] have demonstrated that during hyperglycemia, elevated lipid metabolites promote vascular injury and interfere with protective processes involving vascular endothelial growth factor (VEGF), platelet-derived growth factor (PDGF), activated protein C (APC), insulin, antioxidant enzymes, and anti-inflammatory factors. Further, diabetes induces oxidative and inflammatory responses that cause leukocyte recruitment and plaque formation, contributing to plaque instability and thrombosis [143].

**Figure 5:**
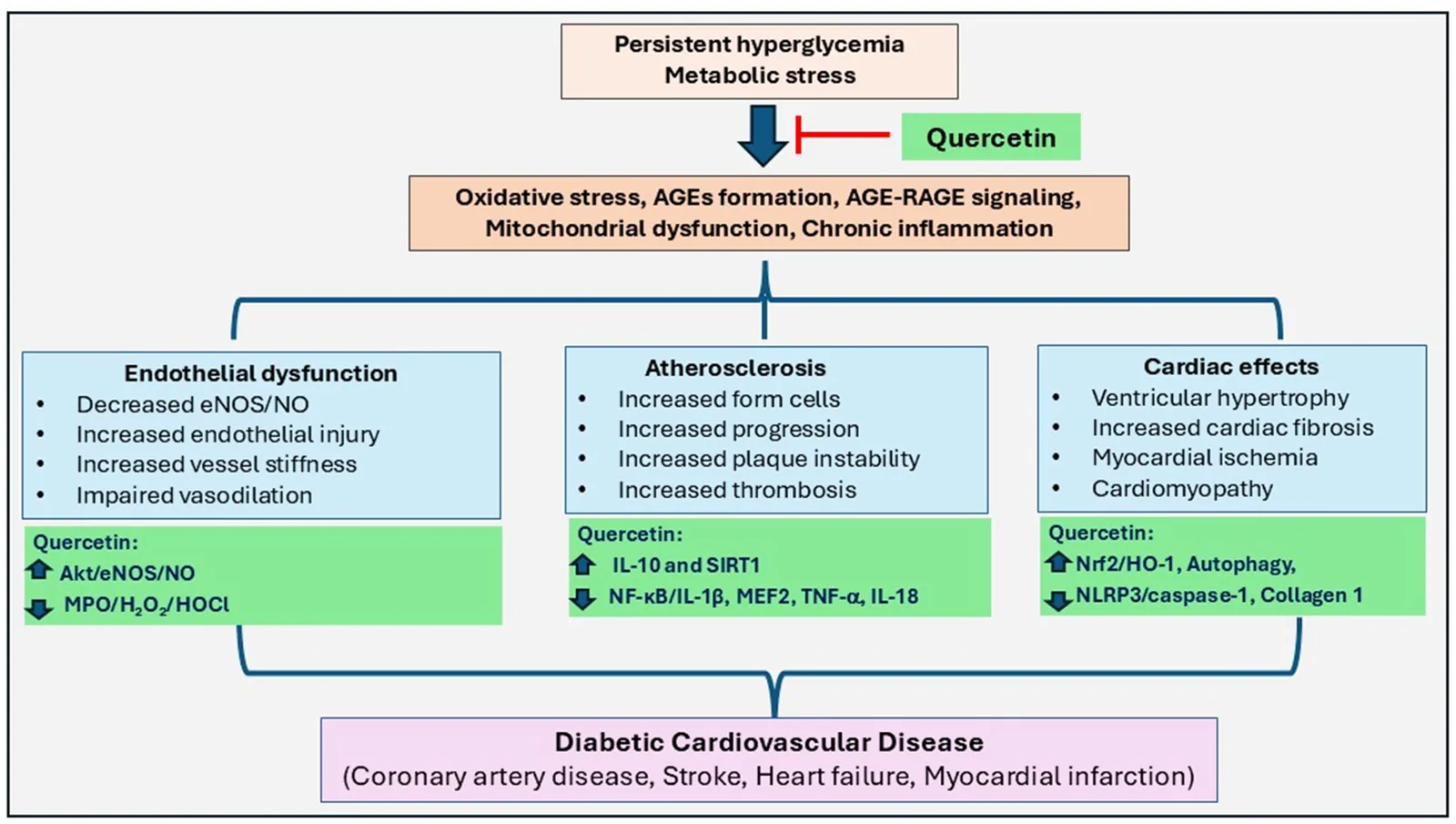
Significance of quercetin in the prevention of diabetic cardiovascular disease. Persistent hyperglycemia causes endothelial dysfunction, impaired eNOS/NO-mediated vasodilation, atherosclerotic plaque formation and instability, thrombosis, ventricular hypertrophy, cardiac fibrosis, myocardial ischemia, and cardiomyopathy. Quercetin could prevent diabetic cardiovascular complications by targeting hyperglycemia-induced oxidative, inflammatory, metabolic, and vascular pathways and preserving endothelial and cardiac function.

Given oxidative stress, inflammation, and endothelial dysfunction in diabetic CVDs, several antioxidants that target these processes have shown therapeutic potential [144,145,146]. Quercetin, with its antioxidant and anti-inflammatory properties, has been shown to prevent diabetic cardiovascular complications [147,148] (Figure 5). Quercetin has been shown to promote antioxidant systems, including glutathione peroxidase, glutathione reductase, and superoxide dismutase (SOD), while inhibiting the production of pro-inflammatory leukotrienes and prostaglandins [149,150]. A few studies also indicate that quercetin can prevent myocardial fibrosis and ventricular hypertrophy [151,152].

Endothelial dysfunction is a major cause of diabetic CVDs [153]. Several studies have particularly examined the effects of quercetin on diabetes-associated endothelial dysfunction. For example, Tian et al. [154] showed that quercetin protected vascular endothelial cells from high glucose-induced injury by suppressing myeloperoxidase (MPO)-dependent hypochlorous acid (HOCl) production. Specifically, they showed that quercetin reduced hydrogen peroxide (H_2_O_2_) generation and acted as a competitive substrate for MPO, thereby limiting MPO/H_2_O_2_-dependent HOCl formation. Further, quercetin also attenuated HOCl-induced endothelial dysfunction in endothelial cells and isolated aortas. In addition, dietary quercetin also improved aortic endothelial function in diabetic mice while reducing vascular MPO expression and activity, suggesting vascular protective effects of quercetin. In another study, Taguchi et al. [155] showed that morin and quercetin promoted vasodilation in diabetic mouse aortas by increasing endothelial nitric oxide synthase (eNOS)-mediated nitric oxide (NO) production through Akt activation. Further, this study also suggests that quercetin may inhibit diabetes-associated endothelial dysfunction through multiple pathways that converge on eNOS activation and NO production. Similarly, Chis et al. [156] showed that quercetin supplementation combined with moderate swimming exercise preserved acetylcholine-induced, NO-dependent endothelial relaxation in rats with type 1 diabetes without altering endothelium-independent relaxation. Together, these data support quercetin’s effect on endothelial NO signaling in diabetes.

Furthermore, Zhang et al. [157] demonstrated quercetin’s vascular effects in diabetes-associated atherosclerosis. They showed that quercetin treatment (30 mg/kg/day) for 2 weeks improved the lipid profile and reduced atherosclerotic lesions, oxidative stress, and lipid peroxidation, while increasing antioxidant enzyme activity in diabetic rats. Quercetin also suppressed inflammation by reducing NF-κB and IL-1β, increasing IL-10, and enhancing SIRT1 expression in the carotid artery. Similarly, Roslan et al. [158] showed that quercetin treatment in diabetic rats reduced fasting blood glucose, increased insulin levels, and decreased cardiac injury markers, including troponin-C, CK-MB, and LDH. They also showed that quercetin - improved cardiac histopathology and reduced oxidative stress, inflammation, and apoptosis by modulating antioxidant enzymes, inflammatory mediators, and apoptosis-related proteins.

Furthermore, a few studies indicate the significance of quercetin in preventing diabetic cardiomyopathy. For example, Bartosova et al. [159] demonstrated that quercetin (20 mg/kg/day) treatment for 6 weeks improved cardiac structure and function in type 2 obese diabetic rats by reversing left ventricular diastolic dysfunction, reducing ventricular wall thickness, and decreasing myocardial collagen accumulation. These changes were accompanied by suppression of several pro-hypertrophic signaling proteins, including MEF2, HDAC4, GATA4, NFAT3, calcineurin, and Erk5, suggesting that quercetin may attenuate cardiac hypertrophy, fibrosis, and pathological remodeling. Similarly, Jiang et al. [160] also showed that quercetin (100 mg/kg/day) improved cardiac function and reduced myocardial fibrosis in a high-fat diet and STZ-induced mouse model of diabetic cardiomyopathy. These effects were associated with suppression of NLRP3 inflammasome signaling, including reductions in caspase-1, IL-1β, and IL-18, as well as decreased expression of the fibrosis-related markers Collagen I and CTGF. The results also suggest that quercetin’s cardioprotective activity may involve both inflammatory and metabolic mechanisms. Another study by Wei et al. [161] showed that quercetin (50 mg/kg/day) promoted the nuclear translocation of Nrf2 and increased the expression of antioxidant proteins, including HO-1, GCLC, and SOD, in a high-fat diet and STZ-induced diabetic rat model. Activation of this pathway prevents cardiomyocyte apoptosis, pyroptosis, inflammation, and cardiac fibrosis. Further, Nrf2 inhibition with ML385 abolished quercetin’s protective effects, supporting a direct role for Nrf2-dependent antioxidant signaling in suppressing pyroptosis and diabetic cardiac injury.

Quercetin has also been shown to regulate the balance between autophagy and apoptosis in the diabetic myocardium. Chen et al. [162] demonstrated that quercetin improved cardiac function and reduced myocardial injury, fibrosis, oxidative stress, and apoptosis in rats with type 2 diabetes. Quercetin restored impaired autophagy by increasing LC3 and Beclin1, reducing P62, and decreasing the interaction between Beclin1 and Bcl-2. These results indicate that quercetin protects against diabetic myocardial injury by promoting autophagy and inhibiting apoptosis. Consistent with this, Ojo et al. [163] showed that diabetes increased mitochondrial permeability transition (mPT) pore opening and mitochondrial lipid peroxidation, resulting in cytochrome c release, activation of caspase-9 and caspase-3, and myocardial lesions. Treatment with quercetin and vitamin E reversed these mitochondrial and apoptotic changes and reduced cardiac damage. These results indicate that inhibiting mPT pore opening and mitochondria-mediated apoptosis may be another mechanism by which quercetin protects the diabetic myocardium. Further, Tan et al. [164] identified IL-6, VEGFA, JUN, MMP9, and EGFR as potential molecular targets in quercetin’s effects on atrial fibrillation (AF). In vivo, quercetin reduced AF duration and atrial remodeling while decreasing p-MAPK expression, suggesting that inhibiting MAPK signaling may enhance its protective actions against AF.

A few studies have also examined the vascular benefits of quercetin in combination with established antidiabetic therapies. For example, Kunasegaran et al. [165] showed that low concentrations of quercetin (10^−7^ M) and pioglitazone (10^−7^ M) synergistically improved endothelial function in diabetic rat aortas by enhancing acetylcholine-induced relaxation and decreasing phenylephrine-induced contraction. The combination reduced superoxide production and increased NO availability, whereas either agent alone at the same concentration produced little effect. Similarly, Chellian et al. [166] reported that combined quercetin and metformin treatment produced greater antidiabetic and vascular protective effects than either treatment alone in STZ-nicotinamide-induced diabetic rats. The combination improved hyperglycemia-associated endothelial dysfunction and normalized eNOS, 3-nitrotyrosine, VCAM-1, CD31, and SIRT-1 expression. These studies suggest that quercetin may complement antidiabetic therapies by improving endothelial function and reducing vascular oxidative stress. Interestingly, supplementing quercetin along with exercise has been shown to provide additional metabolic and cardiac benefits. Khajehlandi and Bolboli [167] showed that quercetin combined with either high-intensity interval training (HIIT) or moderate-intensity continuous training (MICT) elevated metabolic and cardiac outcomes in obese diabetic rats. Quercetin and the exercise regimen reduced blood glucose and improved inflammatory markers and lipid profiles, while both exercise protocols modulated PI3K/AKT, FOXO3, and caspase-8 signaling associated with cardiomyocyte apoptosis.

Despite these outcomes, quercetin’s cardiovascular effects are not uniformly beneficial and may depend on age, disease progression, and the specific cardiac outcome examined. For example, Ferenczyova et al. [168] found that quercetin (20 mg/kg/day) reduced systolic blood pressure and improved vascular relaxation in 6-month-old Zucker diabetic fatty rats, whereas these benefits were absent or reversed in 1-year-old diabetic rats. Quercetin also failed to protect against ischemia–reperfusion injury and worsened post-ischemic recovery in older hearts. Although quercetin altered eNOS and PKCε expression, impaired activation of the PI3K/Akt pathway was associated with a lack of cardioprotection, suggesting that age and diabetes progression may modify the cardiovascular response to quercetin. Similar limitations have been observed in other studies using aged diabetic animals. Boťanská et al. [169] reported that impaired cardiac function in aged obese Zucker diabetic fatty rats was associated with increased MMP-2, decreased MMP-28, and reduced SOD activity. Quercetin treatment (20 mg/kg/day) for 6 weeks did not improve cardiac function or reverse the diabetes-associated alterations in MMP-2 and MMP-28, although it activated Akt signaling and restored SOD-mediated antioxidant defenses. Similarly, Duľová et al. [170] showed that quercetin increased SOD and CAT activities and GPx4 expression, reduced LDH activity, and modulated the Bax/Bcl-2 ratio in diabetic ZDF rats. However, quercetin failed to normalize impaired mitochondrial respiration. These studies thus suggest that quercetin-mediated enhancement of antioxidant defenses does not necessarily translate into restored cardiac function, particularly in aged diabetic animal models.

Recent evidence thus suggests that chronic hyperglycemia-induced ROS generation and inflammatory signaling could cause endothelial dysfunction, atherosclerosis, cardiac remodeling, and cardiomyocyte death. Preclinical studies suggest that quercetin could prevent diabetic CVD by regulating oxidative, inflammatory, metabolic and mitochondrial apoptotic pathways. Clinical studies are needed to determine the conditions under which quercetin may be most effective and whether its use alone or in combination with pharmacological therapy can meaningfully reduce the progression of diabetic cardiovascular disease.

## 6. Quercetin in Diabetic Cataracts

Recent epidemiological studies show that the prevalence of diabetes-induced visual impairment is increasing globally [171,172]. However, the incidence rate of visual impairment in people with diabetes is still up to three times higher than in those without diabetes [173]. Lens opacification and subsequent diabetic cataract are among the earliest, most serious, and most common secondary complications of diabetes mellitus [174,175]. Further, activation of the aldose-reductase-mediated polyol pathway [176,177], hyperglycemia-driven oxidative stress [178,179], accumulation of AGEs [180], and osmotic imbalance [181] have been shown to be involved in the pathophysiology of diabetic cataractogenesis.

**Figure 6:**
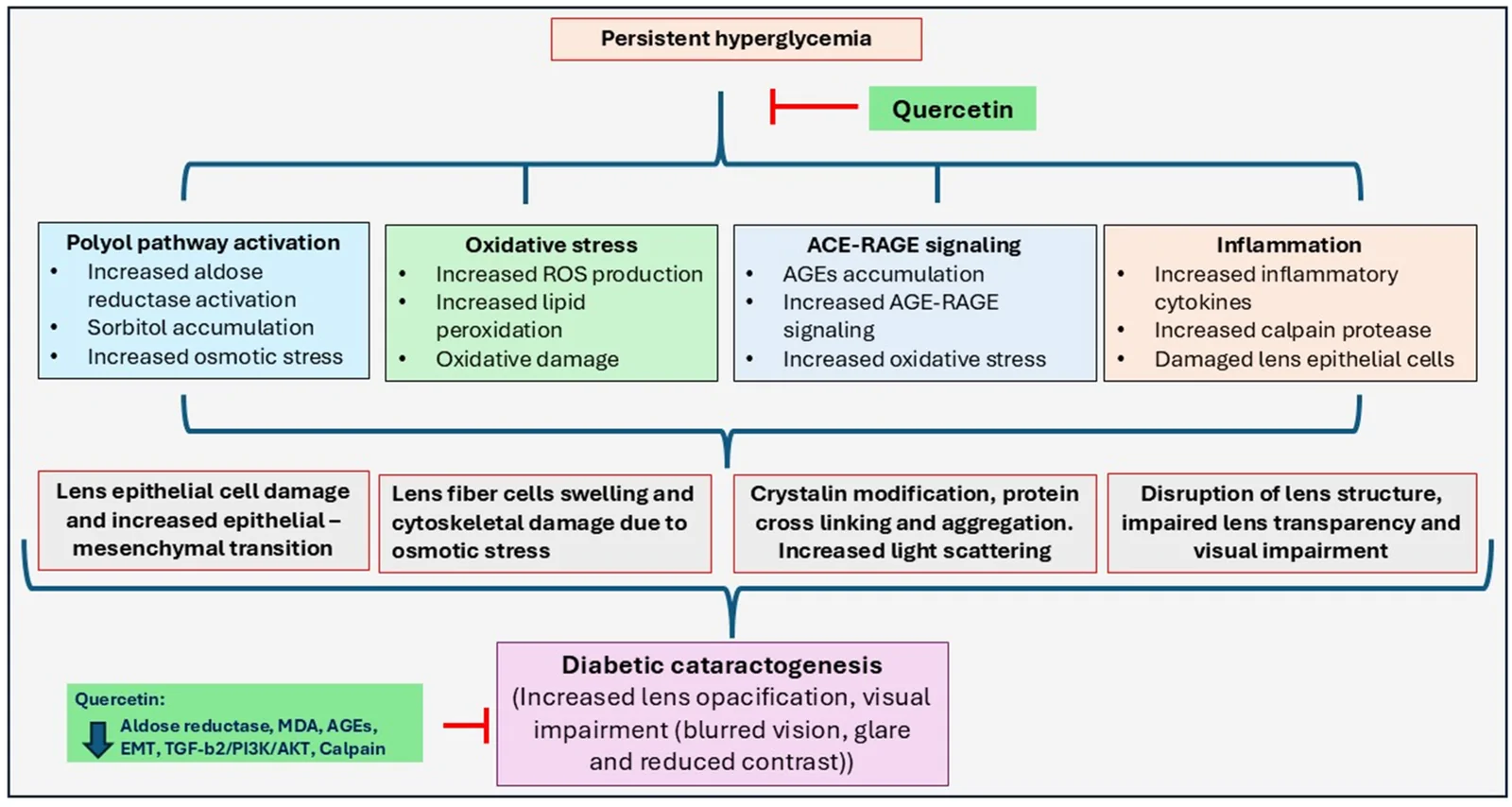
Possible protective role of quercetin against diabetic cataractogenesis. Persistent hyperglycemia, by activating the aldose reductase-mediated polyol pathway, oxidative stress, and AGE–RAGE signaling, causes sorbitol accumulation, osmotic stress, and lens epithelial cell injury. These pathways further contribute to epithelial–mesenchymal transition, lens fiber cell swelling, cytoskeletal damage, crystallin modification, protein cross-linking, and aggregation, which ultimately result in diabetic cataractogenesis and visual impairment. Quercetin could attenuate diabetic cataract development by preventing hyperglycemia-induced osmotic and oxidative pathways.

Although relatively few studies have examined the significance of quercetin and other flavonoids in the prevention of diabetic cataractogenesis [182,183], available evidence suggests that quercetin may act on several mechanisms involved in lens opacification (Figure 6). Stefek and Karasu [184] demonstrated that quercetin has potential as a nonsurgical therapeutic approach because it can target multiple mechanisms involved in cataract development. Its protective effects may involve reducing oxidative stress and glycation, modulating the polyol pathway and calpain proteases, and influencing lens epithelial cell signaling. More recently, Medoro et al. [185] indicated that quercetin may have broader therapeutic potential in age-related ocular diseases, including cataract, diabetic retinopathy, glaucoma, and age-related macular degeneration. This is because of quercetin’s potential serotherapeutic, antioxidant, anti-inflammatory, neuroprotective, and anti-angiogenic effects. These protective mechanisms include targeting cellular senescence, reducing oxidative stress, and regulating inflammatory processes associated with ocular aging and visual impairment.

A few preclinical studies provide evidence of quercetin’s protective effects against diabetic cataractogenesis. For example, in an STZ-induced type 1 diabetic rat model, Lu et al. [186] showed that Ginkgo biloba extract (GbE), rutin, and quercetin delayed cataract progression by reducing aldose reductase (AKR1B1) activity, MDA, and AGE levels while increasing glutathione (GSH). Among the three treatments, quercetin showed the greatest ability to delay diabetic lens opacification. Its protective effect was attributed primarily to inhibition of AKR1B1 activity, lipid peroxidation, and AGE formation.

Additional studies have also identified signaling pathways through which quercetin could protect lens epithelial cells under hyperglycemic conditions. Quercetin suppressed epithelial–mesenchymal transition (EMT) associated with diabetic cataract, and these protective effects were linked to inhibition of the TGF-β2/PI3K/Akt signaling pathway [183]. Similarly, He et al. [187] showed that high glucose exposure increased Aquaporin 1 (AQP1) expression and altered its localization in human lens epithelial SRA01/04 cells by activating the PI3K/Akt/mTOR signaling pathway. They have shown that quercetin (2, 4 and 8 μM) reduced Akt and mTOR phosphorylation and modified AQP1 membrane localization in a dose-dependent manner. These findings suggest that regulating AQP1 translocation by inhibiting PI3K/Akt/mTOR signaling represents another mechanism by which quercetin may protect against diabetic cataract.

Collectively, a few preclinical studies show that quercetin targets multiple mechanisms involved in diabetic cataractogenesis, such as AR activation, oxidative stress, and AGE formation. These studies could support the potential therapeutic use of quercetin to delay diabetic cataract progression. However, additional clinical studies are required to better understand its role in diabetic cataractogenesis.

## 7. Conclusions and Future Perspectives

Diabetes mellitus remains one of the leading causes of microvascular and macrovascular complications worldwide [188,189]. Uncontrolled hyperglycemia causes oxidative stress, inflammation, mitochondrial dysfunction, and advanced glycation end product (AGE)-mediated signaling, which can lead to the development of several diabetic complications such as diabetic retinopathy, nephropathy, neuropathy, cardiovascular disease, and cataracts. Several studies indicate a need for novel, natural therapeutic strategies that target multiple pathogenic pathways involved in the secondary diabetes complications [190,191,192].

Recent studies suggest that quercetin has pleiotropic actions, including antioxidant, anti-inflammatory, anti-apoptotic, and anti-fibrotic effects, that could help prevent and manage diabetes complications. In several animal models of diabetes complications, quercetin has been shown to prevent ROS production, NF-κB-mediated inflammatory signaling, and AGE-RAGE activation, and promote endogenous antioxidant defenses and mitochondrial function. These pleiotropic effects suggest that quercetin functions as a multi-target therapeutic agent that can simultaneously modulate several molecular pathways involved in diabetic complications (Table 1). Although cell culture and animal studies show favorable effects of quercetin on oxidative stress, inflammation, glycemic control, and cardiovascular risk factors, no large clinical trials have demonstrated the significance of quercetin alone in preventing diabetes complications. However, some clinical studies suggest that combining quercetin with other pharmaceutical agents may enhance efficacy in controlling complications. Moreover, current clinical evidence remains very limited, and extensive investigations are needed to understand its clinical significance when used alone and in combination. Further, several heterogeneous studies are needed with different quercetin formulations and dosing regimens.

Even though quercetin has promising antioxidative, anti-inflammatory, and cytoprotective properties, its clinical use is greatly restricted by its poor solubility in water, variable absorption in the gastrointestinal tract, extensive first-pass metabolism, and the low level of the unmetabolized parent compound that reaches the systemic circulation [193,194]. After it is given by mouth, quercetin is extensively bio-transformed in phase II in both the intestinal lining and the liver, mainly by means of glucuronidation, sulfation, and methylation, so that it is the metabolites that end up in the bloodstream rather than significant amounts of free quercetin [195,196]. Studies on quercetin pharmacokinetics in humans show large variability in absorption and metabolism, influenced by quercetin’s position in the body [194]. Because of these pharmacokinetics, serious questions remain about the relevance of in vitro findings to clinical practice; these studies often use concentrations of unconjugated quercetin that are unlikely to be reached in the bloodstream after ordinary oral administration [193]. Furthermore, quercetin metabolites may have different biological activities and distributions compared with the parent compound. Thus, it is difficult to interpret experimental results and determine which molecular species are responsible for the therapeutic effects [197]. To overcome these limitations, researchers have examined various formulation methods, including phospholipid-based delivery systems, cyclodextrin complexes, and encapsulation techniques [198,199,200]. Indeed, a recent systematic review and meta-analysis of human intervention studies by Liu et al. [201] showed that certain formulations lead to a considerable increase in quercetin bioavailability, although greater systemic exposure does not necessarily mean improved clinical efficacy. However, further research on pharmacokinetic–pharmacodynamic relationships, the biological activities of circulating metabolites, tissue-specific distribution, and therapeutic concentrations is needed before quercetin can be confidently used in therapeutic interventions for secondary diabetic complications.

Furthermore, there is a significant gap between the concentrations that can be achieved in the body and those that are typically used in experimental animal studies. Therefore, additional translational studies are needed to support quercetin for clinical use. For example, studies on human pharmacokinetics show that after dietary intake or through conventional oral supplementation, the various quercetin-related compounds in the bloodstream generally reach concentrations in the nanomolar to low-micromolar range. In fact, a maximum plasma concentration of about 0.43 µM was observed after taking 150 mg/day of quercetin [202], and higher-dose preparations have resulted in total plasma concentrations that are in the low-micromolar range [203]. Generally, quercetin in circulation is mainly present as its glucuronidated, sulfated, and methylated metabolites rather than as the unconjugated parent compound. These levels are much lower than the 10 to 100 µM concentrations that are commonly used in in vitro studies [41,46,47]. Moreover, reliable information on the doses of quercetin and its metabolites in human retinal, renal, neural, and cardiac tissues remain very limited. Thus, although preclinical studies suggest that quercetin supplementation may protect against secondary diabetic complications, additional clinical studies are needed to determine physiologically relevant concentrations, tissue-specific bioavailability, and metabolite activity.

Furthermore, to translate preclinical findings into clinical practice, additional randomized controlled trials and safety studies are needed throughout multiple complications of diabetes. Additionally, to increase quercetin’s therapeutic potential, studies on novel and advanced nano-delivery systems are needed to improve its bioavailability. Similarly, combination studies of quercetin with established therapies involving sodium-glucose cotransporter-2 inhibitors, glucagon-like peptide-1 receptor agonists, and metformin could also yield better therapeutic approaches in preventing diabetes complications. Thus, recent studies suggest that quercetin could be a credible adjunctive, multi-target therapeutic candidate in reducing the burden of diabetic complications. However, additional clinical studies are required to understand how quercetin could improve the quality of life of individuals living with diabetes.

## Figures and Tables

**Table 1:** Molecular targets and cellular effects of quercetin in secondary diabetic complications.

Diabetic Complication	Mechanistic Action	Key Molecular Targets	Major Protective Effect	References
Diabetic retinopathy	Neuroprotection	Increase BDNF, NGF, synaptophysin	Protects retinal neurons	[40]
	Inflammation and barrier integrity	Decreases VEGF, AKT/MAPK/NF-κB	Reduces inflammation and preserves retinal barrier	[41]
	Angiogenesis	Decreases MMP9, VEGF	Reduces edema and vascular injury	[42]
	Antioxidant defense	Promotes HO-1	Enhances antioxidant protection	[40,45]
Diabetic nephropathy	Fibrosis and podocyte injury	Decreases TGF-β1/Smad2/3; increases nephrin, podocin	Protects podocytes and reduces fibrosis	[81]
	Apoptosis	Decreases EGFR/ERK1/2, Bax/caspase-3	Reduces podocyte apoptosis	[82]
	Autophagy	Increases FoxO1/STX17	Restores autophagic flux	[83]
Diabetic neuropathy	Mitochondrial function	Activates AMPK/PGC-1α/SIRT1	Improves mitochondrial function	[124]
	Neuroinflammation	Inhibits TLR4/MyD88/NF-κB, NLRP3	Reduces neuroinflammation	[125,126]
	Axonal regeneration	Activates Slit-2/Netrin-1	Promotes axonal repair	[130]
Diabetic cardiovascular disease	Endothelial function	Increases AKT/eNOS/NO	Improves vasodilation	[155]
	Cardiac remodeling	Decreases MEF2, HDAC4, GATA4, NFAT3, calcineurin, NF-kB, NLRP3	Reduces cardiac hypertrophy, inflammation	[157,158,159,160]
	Antioxidant defense	Increases Nrf2, HO-1, GCLC, SOD	Reduces oxidative stress and pyroptosis	[161]
Diabetic cataract	Polyol pathway and glycation	Inhibits aldose reductase, MDA, AGEs	Reduces oxidative damage and glycation	[186]
	Lens epithelial signaling	Inhibits PI3K/Akt/mTOR; altered AQP1 localization	Protects lens epithelial cells	[183,187]

## Data Availability

No new data were created or analyzed in this study. Data sharing is not applicable to this article.
